# The New York State Medical Society and Ethics

**Published:** 1882-04

**Authors:** 


### Editorial.

### ARTICLE XXXI.

### THE NEW YORK STATE MEDICAL SOCIETY AND ETHICS.

Most of our readers have probably learned before this time that at the annual meeting of the Medical Society of the State of New York, in February last, a revised code of ethics was adopted for the government of that society, and of such county medical societies as were eligible to representation in the State society. The revised code was reported by a committee appointed the year previous, and consisted substantially, and to a great extent literally, of those parts of the code of the American Medical Association which relate to "the relations of physicians to the public," and "the relations of physicians to each other," with "the rules governing consultations."

Under the two first heads there is nothing in the revised code that in the least contravenes the letter or spirit of the national code. But under the head of "rules governing consultations" there are three important deviations from the code of the American Association. The first two are contained in the following paragraph, which has been substituted for section 1 of Art. IV, of the duties of physicians in regard to consultations:

"Members of the Medical Society of the State of New York, and of the medical societies in affiliation therewith, *may meet in consultation legally qualified* practitioners of medicine. Emergencies may occur in which all restrictions should, in the judgment of the practitioner, yield to the demands of humanity."

If the reader remembers that in New York, as in this State, the words "*legally qualified practitioners*" mean any and all persons who have received a license from the State Board, whether they call themselves homœpaths, eclectics, hydropaths, or some other *paths*, he will see at once the leading object of the *revisors.* The adoption of the legal qualification or simple State Board license as the standard, and the omission of the clause declaring the unfitness of those "whose practice is based on an exclusive dogma," open the way fully for unrestrained consultations with those practicing every species of *pathy* and *ism* extant. The third deviation from the national code is embraced in the following paragraph: "In case of acute, dangerous or obscure illness, the consulting physician should continue his visits at such intervals as may be deemed necessary by the patient or his friends, by *him*, or by the attending physician." Here is a latitude given for the continued attendance of a consulting physician after having once been called, which should certainly satisfy the most aggressive consultant in New York, or elsewhere.

He is not to be guided in the repetition of his visits by the request of the attending physician, as has been hitherto deemed honorable and just; nor yet by the request of the patient or his friends; but he is to go as often as his own judgment, ambition, or avarice may dictate. It would be difficult to construct a rule more unjust to the attending physician, more open to the grossest abuses, or better calculated to deter the attending physician from suggesting council in the first instance.

We cannot but regard this attempt on the part of the New York State Society to encourage its members to repudiate some of the more important features of the national code, by opening the door to free intercourse with and recognition at the bed-side of every exclusive dogmatist who may be able to procure a license from a State or county board of examiners, or from some kind of chartered medical institution, as most unwise, ill-timed, and injurious both to the interests of the profession and of the community. It is unwise, first, because no good can come to the sick from the introduction of discordant elements into consultations at the bed-side; second, because it directly encourages the practice of hypocrisy and fraud, just where strict integrity and faithfulness is most needed; for either the exclusive dogmatist or the rational physician must consent to dissemble his real convictions and agree to carry out views and practices in which he has no faith, or there can be no agreement; and third, because the step taken is directly calculated to encourage and perpetuate in the minds of the non-professianal public the gross and radical error that legitimate medicine is only *one* of the numerous schools, creeds, or dogmas, distinguished from the rest chiefly by having a more numerous following.

The action of the New York Society was exceedingly ill-timed, because it was taken when it was apparent to every well informed observer, that the followers of Hahnemann were rapidly abandoning their adherence to the distinctive features of homœopathy, and in numerous instances, dropping their distinctive name; and it only required the maintenance of an unbroken adherence to the time-honored and just ethical rule that "no one can be considered a fit associate in consultation, whose practice is based on an exclusive dogma, to the rejection of the accumulated experience of the profession," to have substantially banished the name of homœopath before the end of another generation. The two principal arguments presented by the advocates of the change were "that on grounds of common humanity, it was wrong to refuse skilled services to suffering when in need," and that "it was illiberal and ungenerous, and inconsistent with the spirit of the age, to withhold professional aid from other practitioners simply because they were of a different creed or belief." Both these rest on assumed fallacies, and lead directly to false inferences. The first assumes that a refusal to recognize and consult with an exclusive dogmatist is necessarily a refusal to extend skilled service to the suffering when needed. The assumption here is too plainly false to need illustration.

If a man gets sick, and *voluntarily* takes refuge behind a polecat, and then sends for me to come to his aid with my professional skill, at the same time keeping himself in such a position that I cannot reach him without getting sprinkled with the odoriferous secretion of the polecat, do I *refuse* to extend skilled services to the suffering, when I say to the sick man, you must sever your relations to the polecat before I can come to your aid, knowing that he can make such severance any moment he chooses? It is not only the right, but the duty of every man capable of rendering skilled services to the suffering, to attach such conditions to the rendering of such services as will both preserve his own honor and ensure from those services the greatest benefit to those on whom they are bestowed.

The pretense that it is illiberal and contrary to the spirit of the age to withhold professional aid from other practitioners, *simply* because they were of a different *creed* or *belief*, is based on the plainly implied fallacy that legitimate medicine of the present day is only a *creed* or *belief*, *differing*, it may be, from other creeds or dogmas, but nevertheless a creed. Here is the fundamental error of all the advocates of the so-called *liberal* policy of the New York revisors. Instead of recognizing the all-important fact that the medical science of the present day is neither a creed nor a bundle of dogmas, but all that part of the domain of general science that relates to a knowledge of the structure and functions of the human body in health and disease, and of those agents and influences capable of modifying such structures and functions, they are constantly using expressions countenancing the public error that legitimate medicine is only one of numerous systems, schools or creeds. They seem to forget that legitimate medicine is inherently and necessarily liberal, neither knowing nor recognizing creeds, sects or isms. Her field is as broad as the domain of human knowledge and the existence of human suffering; and whoever chooses to enter that field under the simple title of doctor of medicine enjoys the most perfect liberty of thought and action. He selects his remedies from any source he pleases, applies them in accordance with any principle his own judgment approves, and in any dose which he thinks best calculated to benefit his patients. Consequently he is in a condition to meet and consult with all who are pursuing the same calling on the same broad platform. But when a man adopts a special and exclusive principle or dogma for his guidance, whether it be the law of *similars* or of *attenuations*; or a particular remedy or class of remedies for the cure of all diseases, he becomes of necessity exclusive and illiberal. By adopting his special dogma, and a distinctive name in accordance therewith, he at once voluntarily severs himself from the great body of medical practitioners, and literally notifies all the world, by the title he assumes, that his practice is regulated by the exclusive dogma indicated in such title. He cannot be liberal enough to meet other physicians on the open field of therapeutic inquiry, without belieing his title and placing himself in the attitude of a dissembler or hypocrite. The distinctive title he assumes must either express his honest convictions concerning the proper method of practice, or it becomes a standing lie to the public.

But the man who assumes a distinctive name indicating his adherence to a special system or dogma, not only voluntarily severs himself from the great body of practitioners, but he necessarily places himself in antagonism to that body, and gives his whole influence to the creation and maintenance of a sect or party which serves to distract public attention, create false impressions concerning the nature of the profession, and to interfere with all proper and just legislation on medical and sanitary interests. In other words, when a man adopts an exclusive dogma and a name indicating it to the public, he voluntarily fences himself within a narrow enclosure of his own construction; and neither the interests of humanity, the cause of true science, nor the honor of the profession require any physician to climb over, creep through, or crawl under the fence he has thus erected, to consult with him for any purpose whatever. And yet this is just what the modern revisors of our code of ethics in New York propose to do. Their position is untenable in every aspect in which it can be viewed, and is not sustained by the action of any other respectable body of medical men in Europe or America. If the followers of exclusive dogmas or their patrons wish for consultations, let them drop their distinctive names, come out of their self-constructed narrow pen, and take their place on the common platform of medical science simply as doctors of medicine and honorable men. Until they do this, no physician can meet or consult with them at the bedside, without compromising his own self-respect, and inflicting a positive injury on the cause of true science and humanity.

